# Editorial: Integration of Machine Learning and Computer Simulation in Solving Complex Physiological and Medical Questions

**DOI:** 10.3389/fphys.2022.949771

**Published:** 2022-07-05

**Authors:** Gary An, Michael Döllinger, Nicole Y. K. Li-Jessen

**Affiliations:** ^1^ Department of Surgery, University of Vermont Larner College of Medicine, Burlington, VT, United States; ^2^ Department of Otorhinolaryngology Head and Neck Surgery, Medical School, Division of Phoniatrics and Pediatric Audiology, University Hospital Erlangen, Friedrich-Alexander-University Erlangen-Nürnberg, Erlangen, Germany; ^3^ School of Communication Sciences and Disorders, McGill University, Montreal, QC, Canada; ^4^ Department of Otolaryngology-Head and Neck Surgery, McGill University, Montreal, QC, Canada; ^5^ Department of Biomedical Engineering, McGill University, Montreal, QC, Canada

**Keywords:** machine learning, computer simulation, complex disease, personalized medcine, high fidelity computational method, multi-scale modeling

## Background

This Research Topic, “*Integration of Machine Learning and Computer Simulation in Solving Complex Physiological and Medical Questions*”, brings together two powerful computational approaches to investigate complex disease processes: the use of high-fidelity, mechanism-based simulation models (MSMs), and the training of artificial neural networks (ANNs) via machine learning (ML) and artificial intelligence (AI). These two approaches represent distinct aspects of the scientific process: ML/AI involves correlation identification/hypothesis generation whereas MSMs provide an in silico means for hypothesis testing and conceptual model verification, with capabilities that can complement and address each other’s limitations. High-fidelity MSMs can contain very large numbers of parameters, which poses challenges to effective parameterization and/or parameter space exploration, and can present prohibitive computational costs in terms of executing simulation experiments. Alternatively, ML/AI approaches are notoriously data-hungry (a considerable issue when dealing with biological data sets that are generally orders of magnitude more sparse compared to other ML applications), are highly limited in terms of testing inferred causal relationships, and are often “black boxes” in terms of interpreting why the ANNs do what they do. This Research Topic brings together work that integrates MSM and ML in a complementary fashion. We have organized these papers in the following general classes of investigation.

## Applications of Integrated ML and MSM in Personalized Medicine

The ostensible goal of the practice of medicine is to treat sick individuals with the right drug and the right time, and be able to have such a treatment regimen for every sick patient. MSMs can serve as “digital twins” of individual patients and provide a means of virtually forecasting their future disease course, or, with future developments, aid in personalizing potential therapies. Implicit in this process is the need to capture disease trajectories over time (i.e., integrating time series data), which challenges data-hungry pure ML approaches, but also requires tuning a simulation model to a specific person’s “parameters.” Kuruvila et al. combined convolutional neural network (CNN) and long short-term memory (LTSM) models to infer a listener’s auditory attention in noisy acoustic environments. CNNs were trained with experimental data of electroencephalography (EEG) and speech spectrograms from speakers. The CNN outputs then parsed to the bidirectional LSTM and the auditory attention to speakers were classified. Their results supported the integration of listener-specific EEG signals into ML-powered hearing aids that will help listeners attend to speech signals in noisy scenarios. Schafer et al. applied physics-based network diffusion models to simulate the propagation of misfolded tau proteins in three brain regions of patients with Alzheimer’s disease. Hierarchical Bayesian Inference models were used to obtain posterior probability distributions for two personalized model parameters, namely, the diffusion coefficient and production rate of tau proteins. Personalized models of tau pathology with capability of predicting tau evolution and their associated cognitive functions would be of great use in creating virtual patient controls for clinical trials. van Duuren et al. combined bi-objective evolutionary algorithm (EA) and an established microsimulation model for personalized colorectal cancer screening. EA was used to find personalized screening policies in minimizing the costs while maximizing the number of Quality-Adjusted Life Years gained. Their study results supported the use of computer models to guide policy making and implementation of personalized colorectal cancer screening.

## Machine Learning as Surrogate Models of Complex Mechanistic Models

Developing “lighter weight” surrogate models of complex MSMs would enhance the computational efficiency of simulation experiments. ANNs, as governed by the universal Approximation Theorem (Hornik et al., 1989) are able to recapitulate any generative function and are therefore appealing means of creating surrogate models. Quetzalcóatl Toledo-Marín et al. applied this principle to partial differential equation (PDE) models of biological diffusion. In this case, there is considerable improvement in performance with the surrogate ANN, which allows for both greater complexity of the MSM and more extensive exploration of possible behaviors with simulation experiments. Alternatively, there are types of MSMs that do not have a readily accessible equation form, primarily agent-based models (ABMs). Larie et al. uses ABM simulation data to create a surrogate ANN, but with certain caveats related to properties often found in biomedical ABMs. Firstly, in contrast to deterministic equation-based models, instead of specific trajectories ANN surrogates of ABMs generate a probabilistic “cone” of future trajectories (ala hurricane path prediction). As such, any attempt to use such surrogate models needs to account for this projected uncertainty with updating to produce a rolling forecast horizon. Secondly, the ANN of the ABM also shows the property of path non-uniqueness, which has implications regarding attempts to “reverse engineer” particular pathway or causal network structures from biological data.

## ML-Based Parameter Space Characterization Methods for High-Fidelity MSMs

Complex medical problems require complex solutions. However, there is a tension between using models simple enough to readily parameterize but do not capture key details necessary for clinical utility versus sufficiently expressive but highly complex models with a host of parameters that may not be accessible experimentally. ML methods have thus been applied to the problem of parameter space characterization and uncertainty quantification (Granato and Li-Jessen, 2020) through Model Exploration (ME) (Ozik et al., 2018) methods, such as Random Forest (Garg et al., 2019). Alarid-Escudero et al. utilized the Extreme-scale Model Exploration with Swift (EMEWS) framework for high performance computing (HPC) enabled ME to characterize how experimentally unidentifiable parameters affected the performance of microsimulation decision models regarding the natural history of colorectal cancer (CRC). Leaving these known factors out of a decision model would lead to an intuitively inferior model, and therefore this group used EMEWS to infer regions of identifiable parameter space that produced clinically relevant alterations in the decision model outputs. A different perspective is presented in the paper by Cockrell & An using genetic algorithms (GA) to calibrate a complex ABM. This study introduces a formal mathematical object, the Model Rule Matrix (MRM), intended to account for the inherent “incompleteness” of any mechanism-based simulation model by accounting for all the possible “missing connections” as model parameters. Therefore, as opposed to “parameter fitting” that attempts to reduce experimental/clinical data variation, this approach expands the range of allowable model parameterizations given real-world observations.

## Conclusion

Future work will invariably continue leveraging the strengths of MSMs and ML to offset their inherent limitations. Moving forward, we note multiple open challenges remain, two of which we briefly note:

• The use of synthetic data is ubiquitous in most non-biomedical applications of ML/AI. This need is even more pronounced given the relative sparsity of biological data. However, given the universal Approximating capabilities of ANNs, care must be taken when generating biological time series data such that the ANN does not only “learn” to the generative model. Therefore, developing means to “hide” the generative model from the ANN is a crucial area of investigation and development. The paper in this Research Topic by Cockrell & An begins to address this issue.
• One main concern regarding the use of ML/AI in biomedicine is the opacity of these systems. “Explainable” or “interpretable” AI is a key research topic in the general AI community. The use of MSMs to generate synthetic data can aid in addressing the transparency issues, as the MSMs are explicitly transparent (by virtue of their programmed structure) and essentially represent the conceptual-symbolic model that many consider a necessary component of next generation AI systems. (Garcez and Lamb, 2020).

We hope that the papers in this Research Topic will help spur additional developments and applications in what we consider to be an essential set of methods to better understand and treat complex medical diseases.
